# Work-related factors affecting the retention of medical officers in the preventive health sector in Sri Lanka

**DOI:** 10.1186/s12960-022-00753-w

**Published:** 2022-06-23

**Authors:** Mahendra Arnold, Dinusha Fernando, Kapila Wickramanayake, Palitha Karunapema, Sepali Wickramatilake, Yamuna Fernando, Chandani Denawaka, Pasyodun Koralage Buddhika Mahesh, Sujeewa Pandithrathna

**Affiliations:** 1 Quarantine Unit, Ministry of Health, 385, Deans Road, Colombo 10, Sri Lanka; 2 Office of Regional Director of Health Services, Puttalam, Madampe, Sri Lanka; 3 Medical Supplies Division, Ministry of Health, Deans Road, Colombo 10, Sri Lanka; 4 Health Promotion Bureau, Ministry of Health, Kinsey Road, Colombo 10, Sri Lanka; 5 Regional Director of Health Services Office, Matale, Sri Lanka; 6 Base Hospital, Panadura, Sri Lanka; 7 MOH Office, Nugegoda, Sri Lanka; 8 Ministry of Health, Deans Road, Colombo 10, Sri Lanka; 9 Regional Director of Health Services Office, Rathnapura, Sri Lanka

**Keywords:** Retention, Recognition, Work schedule, Remuneration, Responsibility

## Abstract

**Background:**

Retention of human resources in the healthcare system, particularly doctors at district level is a great challenge faced by the decentralized health systems in poorly resourced countries. Medical Officers of Health (MOH), medical doctors who provide preventive health services, are a particularly important human resource in the preventive health sector in Sri Lanka. This study explores the relative importance of different factors affecting the retention of MOHs in the preventive health sector of Sri Lanka.

**Methods:**

A descriptive cross-sectional study was carried out among Medical Officers of Health in the Colombo district with 18 MOH Offices with 74 medical officers. A pre-tested self-administered questionnaire was used as the study instrument. Data were analyzed using descriptive statistics, correlation and regression analyses.

**Results:**

Of the 74 medical officers 64 responded with a response rate of response rate of 86.5%. Regression analysis showed that all four variables; recognition, work schedule, remuneration and responsibility are positively and significantly correlated with retention of Medical Officers of Health in the preventive health sector. The variable ‘work schedule’ showed the highest impact on the retention of Medical Officers of Health.

**Conclusions:**

In order to retain trained Medical Officers of Health in the Sri Lankan preventive health sector, health authorities should address the factors identified in this study. If policymakers fail to address these factors, preventive health services will face negative implications due to the shortage of key service providers.

**Supplementary Information:**

The online version contains supplementary material available at 10.1186/s12960-022-00753-w.

## Background

Retention of human resources in the healthcare system, particularly medical doctors at district level is a great challenge faced by the decentralized health systems in poorly resourced countries such as Sri Lanka [1]. In Sri Lanka, healthcare services are provided mainly by the government sector and consist of both curative and preventive services. Healthcare institutions range from primary health care institutions at the community level to Outpatient Department (OPD) treatment services in tertiary care hospitals with major specialties and sub-specialties. The healthcare service network spreads across the country and includes the grass root level where domiciliary healthcare services are also provided [2].

Preventive healthcare services are considered as the backbone of the healthcare services in the country. The main emphasis of the preventive sector is to prevent diseases from occurring and thereby minimizing the burden to which the curative sector would otherwise be subjected [3]. These services are provided at the community level by the Medical Officers of Health (MOH), medical doctors with additional preventive health care. In Sri Lanka, preventive health services are geographically divided into MOH areas. MOHs are responsible for playing a vital role in preventing diseases and promoting health at the community level in their respective MOH area [3, 4]. The services provided by a MOH can include: immunization for disease prevention, disease control programs (such as dengue, leptospirosis, malaria, and filaria), maternal and child health services, services for youth and elderly, school health services, food safety, environmental health services, occupational health services, well women services, prevention programs for non-communicable diseases and health promotion. [4] The functions of the MOH area mainly depend on the performance of the MOH as they not only function as service providers, but also as technical experts in preventive health, and as supervisors and managers of other technical staff such as Public Health Inspectors, Public Health Midwives and Public Health Nurses.

Curative healthcare institutions (Hospitals) and MOH Offices can be located in the same geographic area; however, the types of services provided by two of these institution categories differ. MOHs can work both in the curative sector work within hospitals and in the preventive sector, rendered mainly in the field/community setting.

In Sri Lanka, it has been found that there is a preference among MOHs to work in the curative health sector attached to in service delivery. The preventive health sector has become less attractive and MOHs currently in this sector tend to leave to the curative health sector [3]. The shortage of doctors and the preventive sector being less attractive, can lead to the shortage in the preventive sector being higher than that of the curative sector. The number of applicants for preventive health posts is low compared to curative health sector posts. For an example, in the Colombo Regional Director of Health Services area which includes the administrative capital of the country, of the approved cadre of 86 MOH, there were only 74 attached to MOH Offices in 2018. Of these 74, 12 had applied/requested transfers to curative care institutions in 2019. Similar situation is present in other parts of the country as well [5]. This can potentially result in a shortage of MOH. The MOH cannot be replaced by other categories of healthcare staff since MOHs are medical doctors who have to perform specific expert medical tasks. If MOHs are not retained in the preventive health sector, there would be a vacuum created and it would be improbable to maintain quality preventive health services at the divisional level. This would ultimately cause a major negative impact on the entire healthcare system.

Retention of productive employees is a major concern of Human Resource (HR) professionals and healthcare executives. It is more efficient from a system perspective to retain a quality employee than to recruit, train and orient a replacement employee of the same quality [6, 7]. There are several predictors of medical doctors willingness to be retained in the preventive healthcare sector—both financial and non-financial. It is a common belief that remuneration is one of the most important determinants of job retention and indeed an attractive compensation package plays a critical role in retaining the employees [8–11]. Recognition is considered as one of the most important factors among non-financial rewards in increasing the retention of employees. Recognition can strengthen the relationship between organization and people. Recognition can be described as the process where employees are rewarded in organizations by different statuses [12]. Through the recognition, employees feel rewarded and motivated [13, 14]. Intrinsic rewards like opportunities for growth lead employees towards high job performance and motivation than extrinsic rewards like salary [15, 16]. The amount of responsibility placed on the employee can have either negative or positive effects on employee-retention [17, 18]. Medical doctors have different work schedules depending on their place of work which can have a big impact on their retention [19, 20].

This study explores the relative importance of different factors affecting the retention of MOHs in the preventive health sector of Sri Lanka.

## Methods

A descriptive cross-sectional survey study was conducted. The study population consisted of all 74 MOHs in the preventive health sector attached to all 18 MOH Offices in the Regional Directorate of Health Services in Colombo district of Sri Lanka. Being a medical doctor attached to the Preventive Health Sector as an MOH was an inclusion criterion. Considering the practical aspect of service provision in the preventive health sector, at least a 6 -months exposure is necessary for a doctor to become well conversant with the system. Therefore, MOHs with less than 6 months service period in the preventive health sector were excluded as the Medical Doctor would have not been sufficiently exposed to possible work-related factors which could influence the retention.

Through a review of the retention literature, four domains including remuneration, recognition, work schedule, and responsibility were selected as factors affecting the retention of medical officers in the preventive health sector [9,11,12,,14,18,20]. A 25-item questionnaire was prepared with expert input to measure these four domains and the MOH’s willingness to retain in the preventive health sector. Following items were included under each domain.

- Remuneration—Satisfaction with the take home salary, overtime payments, holiday pay, travelling allowance, subsistence allowance (5 questions).
- Recognition—General recognition of the post, recognition by patients, recognition by the community, recognition by family members, recognition by the medical profession (5 questions).
- Work schedule—Satisfaction with present duty hours, working on Saturdays, not required to work on public holidays, not having to do on-call duties, not having to do night duties (5 questions).
- Responsibilities—Administrative responsibilities, responsibilities in clinics, responsibilities in field health services, responsibilities towards the community, responsibilities when off duty (5 questions).
- Willingness to retain in the preventive health sector—Preference to continue in the preventive health sector until completion of present 4-year term, preference to continue in the preventive health sector in the next annual transfer, preference to get transferred to curative sector at the end of present term, preference to get transferred to curative sector before the end of present term, recommend others to serve in the preventive sector (5 questions).

In addition, the questionnaire contained additional 8 questions to obtain information of demographic and service related data.

Primary data were collected through a pre-tested self-administered questionnaire. Pretesting was carried out among 10 MOHs working in MOH Offices in the Puttalam district. The internal consistency of the questionnaire was found to be satisfactory in the pre-test [21, 22]. The questionnaires were then administered in person at MOH Offices. Since MOHs duties primarily involve field work, the questionnaires were also administered through mail where MOHs were not available in the Office at the time of data collection. A coding system was adopted to conceal the identity of the respondent and hence the free expression of participants’ actual attitudes and opinions was facilitated. This potentially improved the overall response rate.

Ethical approval was obtained from the Ethics Review Committee of the National Institute of Mental Health, Sri Lanka.

Domain scores were calculated for the willingness for retention, remuneration, recognition, work schedule and responsibility. Correlation analysis was carried out to see the association between these domain scores with non-parametric correlation coefficient analysis. The following hypotheses on retention of medical officers in the preventive health sector were formulated:

- H1: Positive and significant relationship exists between remuneration and retention.
- H2: Positive and significant relationship exists between recognition and retention.
- H3: Positive and significant relationship exists between work schedule and retention.
- H4: Positive and significant relationship exists between responsibility and retention.

Multicollinearity of the remuneration score, recognition score, work schedule score and responsibility scores was assessed with variance inflation factor (VIF) and tolerance statistics. In the absence of multicollinearity, multivariable analysis was undertaken with multiple linear regression getting all these four variables as independent variables with purposeful selection. The retention score was entered as the dependent variable. The model significance was evaluated with 5% significance level.

## Results

The questionnaire was administered to all 74 eligible MOHs and 64 responded with a response rate of 86.5%. Out of the respondents, majority (62.5%) were males. This was in line with the proportion of MOHs who are males (i.e., 60.8%). Nearly half of the participants (i.e., 48.4%) were between 31 and 40 years of age. The most frequent total service period (of 26.6% of participants) was between 6 and 10 years. It was similar to 25% of MOH population having service period between 6–10 years.

The outputs of the correlation analysis are shown in Table 1. The correlation coefficients of ‘remuneration’, ‘recognition’, ‘work schedule’, and ‘responsibility’ showed positive relationships and were statistically significant. The strongest positive significant association was shown between’recognition and retention’ with a strength of 0.547 at a 0.001 significant level. (*r* = 0.547, *p* < 0.001). A positive and a statistically significant correlation exists between ‘responsibility and retention’ (*r* = 0.487, *p* < 0.001), ‘remuneration and retention’ (*r* = 0.439, *p* < 0.001), ‘work schedule and retention’ (*r* = 0.422, *p* = 0.001).

**Table 1:** Correlation matrix between attitude scores with spearman correlation coefficient

	Recognition	Work schedule	Remuneration	Responsibility	Retention
Recognition	*r* = 1.00 *p* = NA	*r* = 0.369* *p* = 0.003	*r* = 0.493* *p* < 0.001	r = 0.615* p < 0.001	*r* = 0.547* *p* < 0.001
Work schedule	*r* = 0.369* *p* = 0.003	*r* = 1.00 *p* = NA	*r* = 0.563* *p* < 0.001	*r* = 0.300* *p* = 0.016	*r* = 0.422* *p* = 0.001
Remuneration	*r* = 0.493* *p* < 0.001	*r* = 0.563* *p* < 0.001	*r* = 1.00 *p* = NA	*r* = 0.302* *p* = 0.015	*r* = 0.439* *p* < 0.001
Responsibility	*r* = 0.615* *p* < 0.001	*r* = 0.300* *p* = 0.016	*r* = 0.302* *p* = 0.015	*r* = 1.00 *p* = NA	*r* = 0.487* *p* < 0.001
Retention	*r* = 0.547* *p* < 0.001	*r* = 0.422* *p* = 0.001	*r* = 0.439* *p* < 0.001	*r* = 0.487* *p* < 0.001	*r* = 1.00 *p* = NA

*Correlation is significant at the 0.01 level (2-tailed)

The VIF values were below 5 and tolerance statistics were above 0.4, which suggest the non-existence of multicollinearity within the data (Table 2).

**Table 2:** Multicollinearity analysis among the four predictors

Variable	Co-linearity statistics	
	Tolerance	VIF
Recognition	0.749	1.335
Work schedule	0.613	1.632
Remuneration	0.551	1.815
Responsibility	0.790	1.266

The regression analysis (Table 3) revealed that the regression coefficients of ‘recognition’, ‘work schedule’, ‘remuneration’ and ‘responsibility’ were all statistically significant at 0.001 level. Thus, all four hypotheses were positive in direction and were statistically significant.

**Table 3:** Multiple linear regression analysis

Variable	Unstandardized coefficients		Standardized coefficients	*t*	Significance
	B	Std error	Beta		
Recognition	0.564	0.128	0.489	4.411	0.000
Work schedule	1.039	0.185	0.580	5.607	0.000
Remuneration	0.535	0.121	0.489	4.418	0.000
Responsibility	0.440	0.113	0.444	3.903	0.000

The regression analysis showed the highest magnitude of 1.039 representing the ‘work schedule’. It indicates that a positive relationship between work schedule (i.e., no duties public holidays, no on-call and night duties) and retention in the preventive health sector. This indicates that the medical officers are satisfied with the current work schedule in the preventive health sector.

The second highest magnitude of 0.564 was for ‘recognition’ (i.e., recognized by the patients, community, family members and others in the medical profession). This indicates that there is a positive relationship between recognition and retention.

A magnitude of 0.535 was seen for remuneration (i.e., take home salary, overtime payments, holiday payments and travelling allowance). This indicates a positive relationship between the level of remuneration and retention.

Responsibility showed a magnitude of 0.440 (i.e., responsibilities at the clinic, field and community work and having to work when off duty if need arises). This reveals a positive relationship between responsibility and retention.

## Discussion

This is the first documented study exploring impact of work-related factors on retention of medical officers in the preventive health sector in Sri Lanka. Based on the results from correlation analysis, it was shown that there is a positive relationship between selected independent variables (recognition, work schedule, remuneration and responsibility) and dependent variable (i.e., retention). Among four independent variables, the strongest positive significant association was seen between recognition and retention. Regression analysis showed that highest magnitude (1.039) represents the work schedule. All four variables showed positive associations with retention in the preventive health sector. The use of a correlational design and multivariate analysis aided in the development of new knowledge and forming hypotheses that could be used to inform policy and further research.

The current study provided support on the positive and significant relationships between compensation and job retention documented in studies by Mabaso [10] and Chaulagain [11]. Policymakers should ensure that compensation is in line with the current market rate or preferably even more to retain the medical officers. However, the findings were not in line with the study by Judge [8] where association between pay and job retention did not significantly correlate. The study concluded that although pay is a motivating factor the employers should realize that being a pay leader is not likely by itself, to result in satisfied workforce.

The findings of the current study support the positive and a significant relationship existed between recognition and retention. This corroborates research by Nel et al. [15] which indicated that intrinsic rewards like recognition leads employees more towards high job performance, motivation and retention. Policymakers when preparing job descriptions and duty lists should take into consideration this important finding and ensure that the medical officers are recognized through their assigned roles and duties.

The current study revealed a positive and a significant correlation between responsibility and retention. In contrast, a study by Ning [18] among 650 full-time nurses employed in six Chinese hospitals showed that amount of work responsibility was a factor contributing for low retention. This may be due to the fact that the studies were on two different staff categories and medical officers are specifically trained to take a higher level of responsibility compared to other health staff categories as well as the components assessed were different. The medical officers are trained to take responsibilities and being assigned responsibilities itself is a self-motivation of being recognized. Policymakers should assign responsibilities coupled with decision-making authority to MOHs who head a team of public health workers at the community level.

The current study revealed that there was a positive and a significant association between work schedule and retention of medical officers. Similarly, a study by Yaseen [20] on factors affecting doctors’ retention level, it was shown that not getting proper work schedule/structure was one of the main factors affecting retention. The health authorities at the central level who are the decision-makers on work schedules for medical officers should take into account of the study findings and prepare work schedules for MOHs without night and on-call duties and public holidays being off days, which were considered as positive factors for retention.

It is generally perceived among the medical officers that those in the curative sector draw a higher financial gain than the preventive sector due to the additions like higher extra-duty payments and holiday-payments. The satisfaction level of medical officers for the variable ‘remuneration’ was lower than the other three independent variables studied. The MOH as per the work schedule, have a lower probability of working on holidays except during special programs like dengue control programs scheduled on public holidays, which is not a regular occurrence. Due to non-availability of night duties and on-call duties, the amount earned as extra-duty payment is also potentially less. However, MOHs who select the preventive health sector would prefer to have off days and duties without night and on-call duties to spend more time with their families despite the take home salary being less than of the medical officers in the curative sector who have to work on public holidays and do night and on-call duties. Unlike the medical officers in the curative sector, MOH being field officers are entitled to travelling and subsistence allowances; these allowances have very low monitory value and have not been revised for many years in line with the current cost of travelling. The health authorities should take serious note of this fact and revise the rates of overtime and other allowances in the preventive health sector to be in par with the payments in the other sectors.

Being the first documented study on factors affecting retention of MOHs in the preventive health sector, the findings advance the knowledge on the retention issues specifically affecting the MOHs in the government healthcare system in Sri Lanka. Since the government healthcare sector is the main healthcare provider, the government fully funds the training of medical undergraduates and postgraduates under the free education system from the state universities. Furthermore, the government healthcare service provision is heavily dependent on MOHs. Hence the new knowledge on factors affecting the retention of medical officers in Sri Lanka would be much useful for the government to improve the retention of medical officers in the preventive health system. The study revealed that the MOHs value being recognized by the patients, community and the medical profession. The remuneration, especially the low rates of overtime and other allowances had a negative impact on the retention of the MOHs in the preventive sector. Work schedule without on-call duties and being off on holidays have a positive impact on the retention of MOHs. The MOHs who lead the public health team at the community level valued being entrusted with responsibilities. The government could address these factors through policy decisions on human resource management of medical officers and making functional changes in the healthcare system.

There were a number of limitations of the study. Data collection from medical officers was difficult due to their busy work schedules and having to collect data in a setting such as clinics where patient care is given. This may have contributed to the observed non-response rate. The study was conducted in the Colombo district. There are many geographic, cultural and economic variability in different districts in Sri Lanka which can influence the factors studied. Hence, the findings cannot be generalized to the entire country. Nearly half of the participants were between 31 and 40 years of age. The younger age group tend to change their jobs. In general, this may also have had an influence on the retention of medical officers in the current position.

## Conclusions

Work-related factors such as ‘recognition’, ‘work schedule’, ‘remuneration’ and ‘responsibility’ have a positive effect on willingness of medical officers to retain in the preventive health sector in Sri Lanka. Among these four factors, the work schedule showed the highest impact on the retention of medical officers. The policymakers in the Ministry of Health of Sri Lanka can take measures to create favorable contexts related to these factors in order to improve retention of medical officers in the preventive health sector.

## Supplementary Information

**Additional file 1. **Questionnaire.

## Data Availability

The datasets used and/or analyzed are available from the corresponding author on reasonable request. The 25-item questionnaire is given as a supplementary material.

## Abbreviations

MOH: Medical Officer of Health
OPD: Out Patient Department
