# Supplementary material for: Work-related factors affecting the retention of medical officers in the preventive health sector in Sri Lanka
Source: Hum Resour Health. 2022 Jun 23;20:56. doi: 10.1186/s12960-022-00753-w (PMC9229420; doi:10.1186/s12960-022-00753-w)
Supplement: Supplementary file 1 — Additional file 1. Questionnaire. [file 12960_2022_753_MOESM1_ESM.docx]

**QUESTIONNAIRE**

Serial No.

Mark “**X**” in the appropriate cage to the extent that you agree or disagree to the given statements.

| Strongly Disagree |
| --- |
| Disagree |
| Neither disagree or agree |
| Agree |
| Strongly Agree |

|  |  | **Strongly Disagree** | **Disagree** | **Neither disagree or agree** | **Agree** | **Strongly Agree** |
| --- | --- | --- | --- | --- | --- | --- |
| 1. | My post of MOH/AMOH is a well recognized post |  |  |  |  |  |
| 2. | As a MOH/AMOH I get due recognition from the patients |  |  |  |  |  |
| 3. | As a MOH/AMOH I get due recognition from the community |  |  |  |  |  |
| 4. | My post is given due recognition by my family members |  |  |  |  |  |
| 5. | My post is given due recognition by others in the medical profession |  |  |  |  |  |
| 6. | I am satisfied with my present duty hours |  |  |  |  |  |
| 7. | I am satisfied with the duty schedule even though I have to work on Saturdays |  |  |  |  |  |
| 8. | I am satisfied that I am not required to work on public holidays |  |  |  |  |  |
| 9. | I am satisfied that there is no *on call duties* as a MOH/AMOH |  |  |  |  |  |
| 10. | I am satisfied that there is no *Night duties* as a MOH/AMOH |  |  |  |  |  |
| 11. | I am satisfied with my take home salary |  |  |  |  |  |
| 12. | I am satisfied with the amount I earn as overtime payments |  |  |  |  |  |
| 13. | I am satisfied with the amount I earn as holiday pay |  |  |  |  |  |
| 14 | I am satisfied with the amount I get as travelling allowance |  |  |  |  |  |
| 15. | I am satisfied with the amount I get as subsistence allowance |  |  |  |  |  |
| 16. | I am satisfied with the level of Administrative responsibilities that I have to undertake as MOH/AMOH |  |  |  |  |  |
| 17. | I am satisfied with the level of responsibilities in clinics that I have to undertake |  |  |  |  |  |
| 18. | I am satisfied with the level of responsibilities in field health services that I have to undertake |  |  |  |  |  |
| 19. | I am satisfied with the level of responsibilities towards the community that I have to undertake |  |  |  |  |  |
| 20. | I am satisfied with having to undertake responsibilities when need arises even when I am off duty |  |  |  |  |  |
|  |  | **Strongly Disagree** | **Disagree** | **Neither disagree or agree** | **Agree** | **Strongly Agree** |
| 21. | I prefer to continue in the preventive health sector until completion of present 4 year term |  |  |  |  |  |
| 22. | I would prefer to continue in the preventive health sector in the next annual transfer |  |  |  |  |  |
| 23. | I would prefer to get transferred to curative sector at the end of present term |  |  |  |  |  |
| 24. | I would prefer to get transferred to curative sector before the end of present term |  |  |  |  |  |
| 25. | I will recommend others to serve in the preventive sector |  |  |  |  |  |
